# Overexpression of dopa decarboxylase in peritoneal dissemination of gastric cancer and its potential as a novel marker for the detection of peritoneal micrometastases with real-time RT–PCR

**DOI:** 10.1038/sj.bjc.6601544

**Published:** 2004-02-03

**Authors:** C Sakakura, M Takemura, A Hagiwara, K Shimomura, K Miyagawa, S Nakashima, T Yoshikawa, T Takagi, S Kin, Y Nakase, J Fujiyama, Y Hayasizaki, Y Okazaki, H Yamagishi

**Affiliations:** 1 Department of Digestive Surgery, Kyoto Prefectural University of Medicine, Kamigyo-ku, Kawaramachi-dori, Kyoto 602-8566, Japan; 2 Genomic Sciences Center, RIKEN,Yokohama Institute, 1-7-22 Suehiro-cho, Tsurumi-district, Yokohama 230-0045, Japan

**Keywords:** DDC, real-time RT–PCR, gastric cancer, peritoneal dissemination

## Abstract

We previously performed a global analysis of the gene expression of gastric cancer cell lines established from metastases to the peritoneal cavity with the cDNA microarray method, which made it possible to analyse the expression of approximately 21 168 genes for the identification of novel markers for the detection of micrometastases in the peritoneal cavity. One of the upregulated genes is dopa decarboxylase (DDC), which is responsible for the synthesis of the key neurotransmitters dopamine and serotonine. We have examined its potential as a novel marker for the detection of peritoneal micrometastases of gastric cancer.

DDC mRNA in the peritoneal wash from 112 gastric cancer patients was quantified for comparison of carcinoembryonic antigen (CEA) mRNA by means of real-time reverse transcriptase–polymerase chain reaction (RT–PCR) with a fluorescently labelled probe to predict peritoneal recurrence. The quantity of DDC and CEA correlated with wall penetration. Real-time RT–PCR could quantitate 10–10^6^ DDC-expressing gastric cancer cells per 10^7^ mesothelial cells. The cutoff value was set at the upper limit of the quantitative value for noncancer patients, and those above this cutoff value constituted the micrometastasis (MM+) group. Of 15 cases with peritoneal dissemination, 13 were MM+DDC (87% sensitivity), and one of 48 t1 cases was MM+ (98% specificity). DDC levels in peritoneal washes from patients with synchronous peritoneal metastases were more than 50 times higher than in those from patients without metastasis (*P*<0.01). For 15 cases of peritoneal dissemination (seven cases were cytologically positive), DDC was positive in 13 cases (87% sensitivity), but CEA failed to detect micrometastases in four cases (73% sensitivity), indicating that DDC is in some cases superior to CEA for the detection of peritoneal micrometastases of gastric cancer in terms of sensitivity as well as specificity, especially for poorly differentiated adenocarcinomas. A combination of CEA and DDC improved the accuracy of diagnosis up to 94%.

These results suggest that DDC is potentially a novel marker for peritoneal dissemination of gastric cancer and that quantitative RT–PCR of DDC is reliable and efficient for the selection of patients for adjuvant intraperitoneal chemotherapy to prevent peritoneal recurrence.

The prognosis for gastric cancer that has invaded as far as the gastric serosa is still poor, with a 5-year survival of less than 35% (Yamazaki *et al*, 1989). Peritoneal dissemination is reported to be the most frequent type of recurrence after curative resection in such gastric cancer cases (Baba *et al*, 1989; Kodera *et al*, 1996). Free cancer cells derived from serosal invasion may thus be an indicator of early peritoneal seeding with subsequent formation of metastatic colonies, so that their detection is likely to be a useful tool for predicting the outcome for patients with advanced gastric cancers (Abe *et al*, 1995; Bonenkamp *et al*, 1996). Conventional intraperitoneal cytology (CY) has recently become a common procedure for the detection of free gastric cancer cells in the peritoneal cavity. Cytology-positive cases are classified as Stage IV of the UICC gastric cancer classification and curative operation is impossible in such cases. Depending on the cytological results, operative procedures including lymphnode dissection may have to be changed, and intensive chemotherapy may have to be used for such cases. Conventional CY, however, lacks sensitivity and some patients with negative CY results have nevertheless been found to show recurrence in the form of peritoneal dissemination. A previous study has indicated the usefulness of carcinoembryonic antigen (CEA)-specific RT–PCR to detect free cancer cells in the peritoneal fluids (Nakanishi *et al*, 1997); CEA is not perfect as a marker, so that identification of more reliable markers is needed.

We previously performed a global analysis of the differential gene expression of a gastric cancer cell line established from a primary main tumour and of other cell lines established from metastases to the peritoneal cavity (Sakakura *et al*, 2002). The application of a high-density cDNA microarray method made it possible to analyse the expression of approximately 21 168 genes. Our investigations showed that 24 genes were upregulated and 17 genes downregulated, in addition to the expression sequence tags (ESTs) in gastric cancer cell lines established from metastases to the peritoneal cavity. One of these upregulated genes is dopa decarboxylase (DDC).

DDC is an enzyme that metabolises DOPA (3,4-dihydroxyphenylalanine) to DA (dopamine), and increased urine excretion of catecholamine metabolites is well known as a key diagnostic feature of neuroblastoma (Eldrup *et al*, 2001). DDC is responsible for the synthesis of the key neurotransmitters dopamine and serotonin, and is frequently expressed in neuroblastoma and small-cell carcinoma of the lung (Jensen *et al*, 1990; Gilbert *et al*, 1999). DOPA is used as a tumour marker for children with neuroblastoma (Ikeda *et al*, 1996), but the role of DDC in gastric cancer peritoneal dissemination is still unclear.

In the study presented here, even more sensitive detection of micrometastases of cancer cells could be achieved through amplification of the novel marker DDC by means of quantitative reverse transcriptase–polymerase chain reaction (RT–PCR) for peritoneal lavage fluid cells. This detection method can be expected to result in a more accurate prediction of peritoneal recurrence in gastric cancer patients.

## MATERIALS AND METHODS

### Cell culture, peritoneal washes and RNA preparation

Gastric cancer cell lines SNU-1, SNU-5, SNU-16, SNU-620 cells were established previously by Park *et al*. KATO-III, GT3TKB, MKN7 and acute myeloid leukaemia cell line HL60 were purchased from the Riken Cell Bank (Tsukuba, Japan), and NUGC-3 cells from the Health Science Research Resources Bank (Osaka, Japan). GT3TKB cells were maintained at 37°C in a humidified atmosphere of 5% CO_2_ in high-glucose DMEM (Sigma, St Louis, MO, USA), whereas MKN7 and NUGC-3 were maintained in RPMI 1640 (Sigma). Both media were supplemented with 10% foetal bovine serum, penicillin and streptomycin. When they had reached 80–90% confluence, cells were washed with ice-cold PBS and homogenised immediately in Isogen reagent (Nippon Gene, Osaka, Japan), and total RNA was extracted. mRNA was extracted from each cell line with the FAST track kit Ver.2 (Invitrogen), according to the manufacturer's instructions. Mesothelial cell line Met5A was established by Reddel *et al*. (Duncan *et al*, 1993; Nakabayashi *et al*, 1999).

The study population consisted of 112 patients with gastric cancer and 15 patients with benign diseases, including cholecystolithiasis and leiomyoma of the stomach, who underwent surgery at Kyoto Prefectural University of Medicine between 1999 and 2003. Ascitic fluid was collected from the Douglas cavity during laparotomy. Written informed consent was obtained from each patient prior to tissue acquisition. In the absence of ascites, 150 ml of saline was introduced into the Douglas cavity at the beginning of the operation and aspirated after gentle stirring. These washes were centrifuged at 2000 rpm for 10 min to collect intact cells, which were rinsed with PBS, dissolved in ISOGEN RNA extraction buffer (Nippon Gene, Tokyo) and stored at −80°C until use. Samples were centrifuged at 12 000 rpm in a rotor (RA-48J, Kubota Fujioka, Japan) at 4°C for ethanol precipitation.

### Northern blot analysis

Northern blot was performed as previously described by us (Sakakura *et al*, 1994, 1996). In brief, the total cellular RNA was prepared with the guanidine isothiocyanate–phenol–chloroform procedure. Poly(A)^+^ RNA was selected with an oligo dT column (Cat. No. R500-50, Invitrogen), and then fractionated on 1% agarose/2.2 M formaldehyde gels. Probes were labelled with ^32^P by random priming. Each blot was then hybridised with the probe for the selected gene and *β*-actin. Signals were analysed with a BAS 2000 image analyser followed by calculation of the degree of overexpression compared to the control.

### Real-time quantitative RT–PCR

cDNA was produced from total RNA by using a Superscript Preamplification System (BRL, Bethesda, MD, USA) in accordance with the procedures suggested by the manufacturer. Total RNA (2 *μ*g) was heated to 70°C for 10 min in 14 *μ*l of duethylpyrocarbonate-treated water containing 0.5 *μ*g oligo (dT). Synthesis buffer (10 ×, 500 mM Tris-HCl, pH 8.3, 750 mM KCl, 30 mM MgCl_2_), 2 *μ*l 10 mM dNTP mix, 2 *μ*l 0.1 M DTT, and reverse transcriptase (Superscript RT; 200 U *μ*L^−1^, GIBCO BRL, Gaithersburg, MD, USA) were added to the sample. The resulting reaction mixture was incubated at 42°C for 50 min, and the reaction was terminated by incubating the mixture at 90°C for 5 min.

Quantitative PCR was performed using the real-time'Taqman TM' technology as described by Nakanishi *et al* (2000) and the results were analysed on a Model 5700 Sequence Detector (Applied Biosystems Corp., Foster City, CA, USA).

The DDC RT–PCR primers were 5′-AAGCACAGCCATCAGGATTCA-3′ and 5′-TGGACATGCTTGCGGATATAAG-3′, and the CEA RT–PCR primers were 5′-TCTGGAACTTCTCCTGGTCTCTCAGCTGG-3′ and 5′-TGAAGCTGTTGCAAATGCTTTAAGGAAGAAGC-3′. Hybridisation probes, which bind to PCR products, were labelled with a reporter dye, FAM, on the 5′ nucleotide and a quenching dye, TAMRA, on the 3′nucleotide. The sequences of hybridisation probes were CEA: 5′-(FAM) CATCTGGAACTTCTCCTGGTCTCTCAGC (TAMRA)-3′ and DDC: 5′-(FAM) AAGCACAGCCATCAGGATTCA (TAMRA)-3′.

In total, 50 *μ*l reactions contained: 1.25 U Amp-Taq DNA polymerase, 1 × PCR reaction buffer, 180 ng of each primer, 200 mM dNTP, 400 mM dUTP, 100 nM Taqman probe and 0.5 U Amplirase (Applied Biosystems Corp.). The Ct value corresponding to the number of cycles at which the fluorescence emission monitored in real time reached a threshold of 10 standard deviations (s.d.) above the mean base line emission from cycles 1 to 40 was measured in serial dilutions of control cDNA, and analysed for each target. CEA and DDC served as standard curves from which the rates of change in Ct values were determined. The cycling parameters were 2 min at 50°C and 10 min at 95°C, followed by 40 cycles of 15 s at 95°C and 1 min at 60°C.

To minimise the errors arising from the validation of the initial amount of RNA in the samples, *β*-actin mRNA was amplified as an internal reference against which other RNA values could be normalised. The primers and the probe for the *β*-actin RNA were purchased from Applied Biosystems. Normalised results were expressed as the ratio of copies of each of the genes to copies of the *β*-actin gene.

### Statistical methods for analysis

Statistical analysis was performed using the NAP system programmed by Aoki (Version 4.0). The first objective of the statistical analysis was to examine the influence of *DDC* expression on clinicopathological factors with the unpaired *T*-test. The various groups of patients were compared by means of either *χ*^2^ test or Mann–Whitney *U*-test. The results with *P*-values of less than 0.05 were considered statistically significant.

## RESULTS

### LightCycler validation of rapid quantitative RT–PCR

Real-time fluorescence PCR monitoring with the LightCycler using hybridisation probes allowed for rapid and sensitive detection of DDC mRNA from the patient samples. With this method, 10–10^6^ DDC-expressing gastric cancer SNU-16 cells per 10^7^ mesothelial cells could be quantitated. No significant level of DDC mRNA was detected in peripheral blood lymphocytes or mesothelial cells from healthy volunteers.

**Figure 1:**
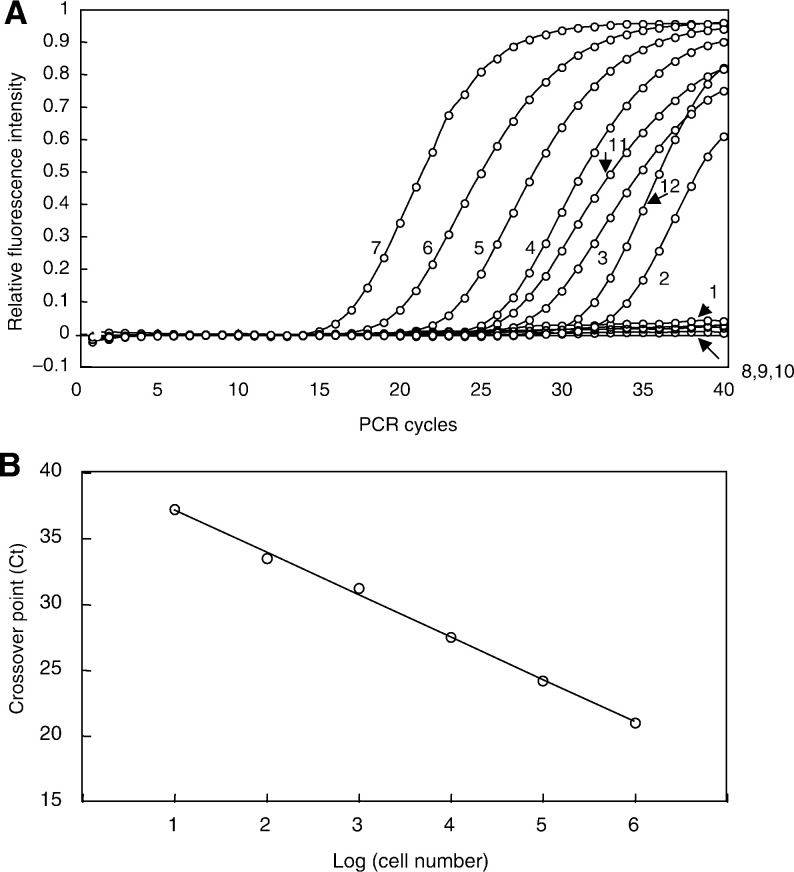
Representative results of real-time RT–PCR with LightCycler and calibration curve for DDC mRNA estimation. (**A**) Run profile *vs* PCR cycles. Six external standards (lines 1–6) were compared with two patient samples (lines 10 and 11) with unknown concentrations, which were amplified with real-time ‘Taqman TM’ technology and analysed with a Model 5700 Sequence Detector: line 1=1; line 2=10; line 3=10^2^; line 4=10^3^; line 5=10^4^; line 6=10^5^; line 7=10^6^ SNU-16 gastric cancer cell equivalent cDNA, line 8=Met 5A; line 9=HL60; line 10=peritoneal wash with negative conventional RT–PCR results; lines 11 and 12=peritoneal wash with positive conventional RT–PCR results. (**B**) Calibration curve for DDC mRNA estimation constructed from data for external controls (**A**) by plotting the crossover points (Ct) against the log (SNU-16 cell number). Relative DDC mRNA values in patient samples were calculated with reference to this curve.

Quantification of messages with the LightCycler was assessed by determination of the crossover point (Ct), the cycle when fluorescence of a given sample rose above the background level to yield the maximal slope with respect to log-linear amplification. Figure 1B

illustrates a standard curve constructed by plotting the log number of 10-fold serially diluted SNU-16 cells against their respective Cts. DDC mRNA values for patient samples with unknown concentration were calculated with reference to the calibration curve.

### Expression of DDC mRNA in gastric cancer cell lines, mesothelial cell line, normal gastric mucosa and cancerous tissues

**Figure 2:**
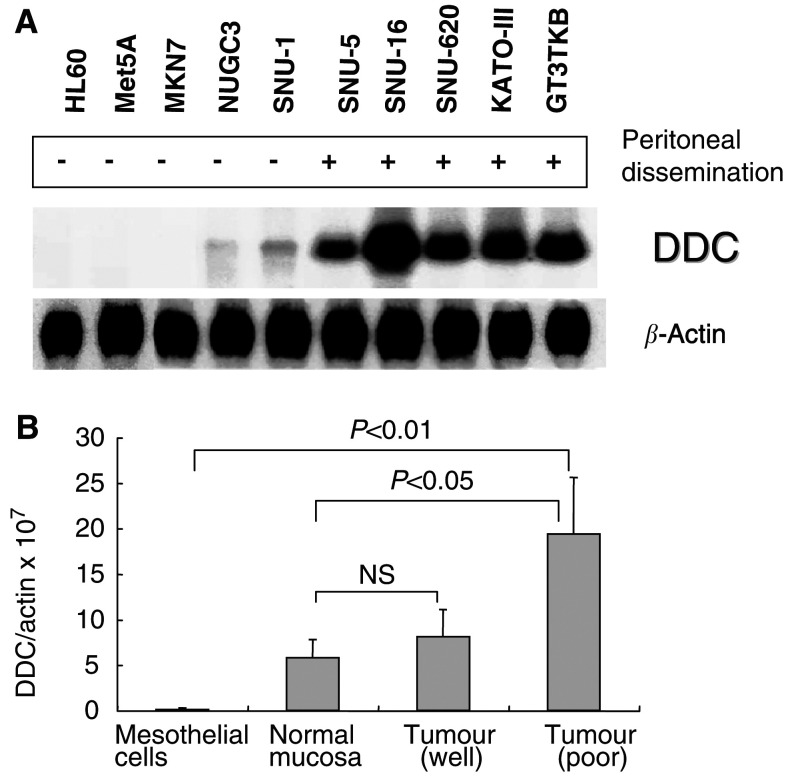
Specificity of DDC expression. (**A**) DDC mRNA expressions in various gastric cancer cell lines were analysed by Northern blotting. Northern blot analysis of differently expressed genes in eight gastric cancer cells. Upregulated genes in gastric cancer cells from malignant ascites compared to those in the primary lesion. (**B**) DDC mRNA expression in primary gastric cancers, normal gastric mucosa and mesothelial cells were analysed by quantitative RT–PCR. Well: well-differentiated adenocarcinonoma; poor: poorly differentiated adenocarcinoma.

Northern blot analysis showed a high expression level of DDC only in cells with a high potential for peritoneal dissemination and a low expression level in all the cells with a low potential. The mesothelial cell line Met5A and human leukaemia cell line HL-60 showed a very low level of DDC expression (Figure 2A

).

The DDC expression was detected in both normal gastric mucosa and gastric cancer tissues of clinical specimens, but the DDC expression was higher in the latter than in the former. It was significant in poorly differentiated adenocarcinomas, but the difference was not significant between cancerous tissue and normal mucosa in cases of well-differentiated adenocarcinoma. The DDC expression was significantly higher than in mesothelial cells from peritoneal wash, especially in the case of poorly differentiated adenocarcinoma (Figure 2B). The summary of DDC mRNA and clinicopathological factors in gastric cancer patients is shown in Table 1

**Table 1:** Summary of DDC mRNA and clinicopathological factors in gastric cancer patients

		**DDC mRNA**		
**Variables**		**Positive**	**Negative**	*P*-value*
Sex	Male	9	44	NS
	Female	11	48	(0.7332376)
Differentiation	Differentiated	4	47	0.002314**
	Undifferentiated	16	45	
Depth of invasion	t1, t2	3	66	0.0073323**
	t3, t4	17	26	
Lymphatic invasion	Negative	3	45	0.0322323**
	Positive	17	47	
Vascular invasion	Negative	11	48	N.S.
	Positive	9	44	(0.067332376)
Lymph node metastasis	Negative	3	42	0.037622**
	Positive	17	50	
Peritoneal dissemination	Negative	2	90	0.0013339**
	Positive	18	2	

t = classification: t1=mucosa to submucosa; t2=muscularis propria to subserosa; t3=serosa-exposed; t4=serosa-infiltrating.

* Mann–Whitney test;

** statistically significant, NS=not significant.

. DDC expression correlates with differentiation, depth of invasion, lymphatic invasion and peritoneal dissemination.

### DDC mRNA/*β*-actin mRNA ratio and the degree of wall invasiveness

**Figure 3:**
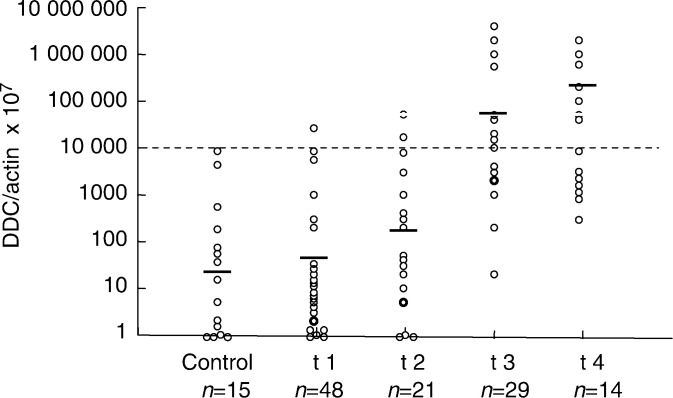
Relative values for DCC mRNA/*β*-actin mRNA ratios in peritoneal washes of gastric carcinoma patients by depth of invasion (pT category). DDC mRNA values correlated with depth of cancer invasion (*P*<0.01).

In order to standardise the amount of RNA in each sample, *β*-actin mRNA was used as an internal control. The value for extracted DDC was determined as the DDC mRNA/*β*-actin mRNA ratio. The average DDC mRNA/*β*-actin mRNA ratio (× 10^7^) by *t* classification was: t1, 39+13; t2, 162+93; t3, 39210+6613; t4, 17300+4139 (average+s.d.). Subjects were further classified into positive cases (t3, t4) and negative (t1, t2) for invasion of the serosa. The results showed that the DDC mRNA/*β*-actin mRNA ratio correlates with the degree of wall invasiveness. The plot for DDC mRNA/*β*-actin mRNA (× 10^7^) is shown in Figure 3

. The DDC mRNA/*β*-actin mRNA ratio was significantly higher for cases positive for invasiveness of the serosa than for negative cases.

### DDC as an independent prognostic factor

**Figure 4:**
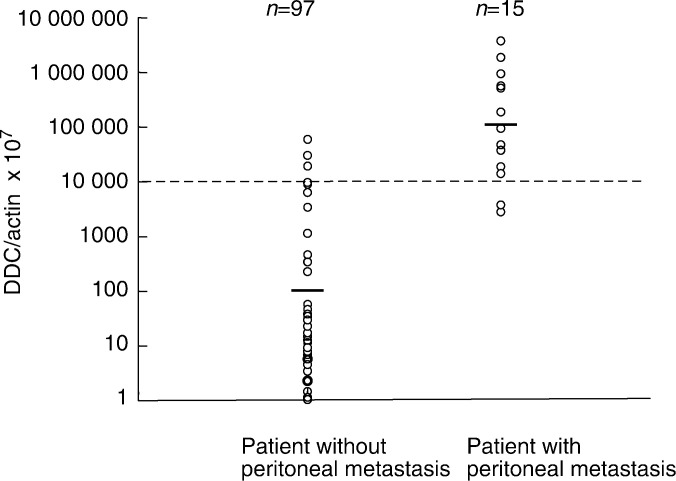
Relative DDC mRNA values for peritoneal washes from the Douglas cavity measured by real-time RT–PCR with the Light Cycler in gastric cancer patients with or without synchronous peritoneal metastasis. DDC mRNA values for washes from metastasis-positive patients were significantly higher than those for metastasis-negative patients (*P*<0.001).

The highest value of the DDC mRNA/*β*-actin mRNA ratio determined for noncancer cases undergoing gastric surgery was adopted as the cutoff value (broken line on the graph), and values greater than this value were regarded as positive (DDC+). Of the total of 112 cases, 20 were determined to be DDC positive, and 15 showed positive cytology (CY+) or were observed to have peritoneal metastases. Since 13 of these 15 subjects had results above the cutoff value, they were classified as DDC+ (86% sensitivity). Moreover, one of the 48 t1 cases was DDC+ (98% specificity) (Figure 4

).

### Comparison of sensitivity and specificity of DDC and CEA as markers for peritoneal micrometastasis of gastric cancer

CEA mRNA/*β*-actin mRNA was also measured in all clinical samples, and the results are presented as the CEA mRNA/*β*-actin mRNA ratio. The results for CEA mRNA/*β*-actin mRNA (× 1000) were: t1, 5.3+3.5; t2, 8.4+6.8; t3, 71.1+22.8; and t4, 643.9+378.3. A correlation was observed between the degree of wall invasion and the level of CEA mRNA/*β*-actin mRNA (data not shown). Moreover, when classified, according to the invasion of serosa into positive (t3, t4) and negative (t1, t2), a significant difference was observed between the two groups (data not shown). The highest value for the CEA mRNA/*β*-actin mRNA ratio in noncancer cases was set as the cutoff value, and the cases showing a value greater than this were regarded as CEA positive. Of the 15 subjects who were also CY+ or were intraoperatively observed to have peritoneal metastasis, 11 were CEA+. However, for four subjects below the cutoff value, (CEA−) metastasis was observed (73% sensitivity), and three of the 48 subjects who were t1 cases were classified as CEA+ (93% specificity) (Tables 2

**Table 2:** Summary of –RTPCR results for DDC/CEA expression and peritoneal wash in gastric cancer

		**No. of positive cases in quantitative RT–PCR**		
**Depth of invasion**	**No. of patients**	**CEA**	**DDC**	**Cytology**
*Primary tumour*				
t1	48	3 (3%)	1 (2%)	0 (0%)
t2	21	2 (9%)	2 (9%)	0 (0%)
t3	29	9 (31%)	10 (34%)	5 (17%)
t4	14	5 (36%)	7 (50%)	2 (15%)
				
Total	112	19 (16%)	20 (18%)	14 (20%)
				
Benign disease	15	0 (0%)	0 (0%)	0 (0%)

classification: t1=mucosa to submucosa; t2=muscularis propria to subserosa; t3=serosa-exposed; t4=serosa-infiltrating.

and 3

**Table 3:** Clinicopathological features of cases with peritoneal recurrence of gastric cancer and quantitative results of DDC and CEA for peritoneal washes

			**Markers**		
**Case no.**	**Stage^a^**	**Histology^b^**	**CEA**	**DDC**	**CEA and DDC**
1	P1H0N2T3CY1 Stage IV	por	+	+	+
2	P0H0N2T3CY0 Stage IIIB	sig	−	+	+
3	P0H0N1T3CY1 Stage IV	por	+	+	+
4	P0H0N1T3CY0 Stage IIIA	tub	+	+	+
5	P1H0N2T3CY0 Stage IIIB	sig	+	+	+
6	P0H1N1T4CY1 Stage IV	sig	+	+	+
7	P0H1N3T3CY0 Stage IV	por	+	+	+
8	P1H1N2T3CY0 Stage IV	por	+	+	+
9	P0H0N2T3CY1 Stage IV	sig	+	+	+
10	P1H0N1T4CY1 Stage IV	por	−	−	−
11	P0H0N2T3CY0 Stage IIIB	muc	+	+	+
12	P0H0N3T3CY0 Stage IV	pap	+	+	+
13	P0H0N1T3CY0 Stage IIIA	sig	+	+	+
14	P0H0N2T3CY1 Stage IV	por	−	+	+
15	P0H0N2T3CY1 Stage IV	por	−	+	+
					
	Sensitivity for the detection of MM		73% (11/15)	87% (13/15)	94% (14/15)

These cases were subsequently found to show recurrence of peritoneal carcinomatosa.

a Clinical stage according to Japanese Gastric Cancer Classification.

b Histology of the primary lesion according to Japanese Gastric Cancer Classification.por=poorly differentiated adenocarcinoma, sig=signet ring cell carcinoma, tub=tubular adenocarcinoma, muc=mucinous carcinoma, pap=papillary adenocarcinoma.

).

Although four subjects (cases 2, 11, 14 and 15 in Table 3) registered a CEA – a result of poorly differentiated adenocarcinoma, they showed evidence of peritoneal metastasis at an early stage. Three of these cases showed positive DDC mRNA/*β*-actin mRNA ratio results (Tables 2 and 3). As shown in Figure 2, DDC is frequently overexpressed in poorly differentiated adenocarcinomas, suggesting that DDC is viable as a novel marker for cases that are undetectable with CEA. As shown in Table 3, the combination of CEA and DDC improved the accuracy of diagnosis up to 94%.

## DISCUSSION

Peritoneal dissemination is the most frequent pattern of gastric cancer recurrence and prognosis for gastric cancer patients with peritoneal dissemination is invariably poor. Some previous reports have indicated that intraperitoneal chemotherapy improves the survival of these patients, but it can sometimes be life-threatening because of side effects. We developed a novel technique to administer the anticancer agent mitomycin-C adsorbed on activated carbon particles (MMC-CH) (Hagiwara *et al*, 1992, 1993a, 1993b, 1999; Takahashi *et al*, 1995). This form of administration can deliver a large amount of the anticancer agent, because the corpuscular particles are not absorbed through the capillary wall. Instead, they are retained in the cavity, which remains closed for a long time and thus can maintain a high concentration of anticancer agents. We previously reported on this new form of drug administration and its therapeutic efficacy for peritonitis carcinomatosa of gastric cancers (Hagiwara *et al*, 1992). However, sometimes side effects occur such as ileus, fever, leukocytopenia and so on, so that it has been necessary to determine the indication for this therapeutic application to avoid such side effects.

The extreme sensitivity of RT–PCR makes it possible to diagnose micrometastases on the basis of tissue-specific mRNA expression of tumour cells in peripheral blood, bone marrow, lymph nodes and cerebrospinal fluid (Burchill *et al*, 1995; Johnson *et al*, 1995; Maehara *et al*, 1996; Noguchi *et al*, 1996; Mori *et al*, 1998). Recently, the use of rapid quantitative RT–PCR-based screening methods for the detection of micrometastasis from clinical specimens has become standard procedure (Nakanishi *et al*, 2000; Sato *et al*, 2001; Oki *et al*, 2002). Many different kinds of molecular markers for the detection of peritoneal micrometastases have been described, such as conventional RT–PCR assay of CEA, keratin 19 and AFP using the peritoneal wash from gastric cancers (Nakanishi *et al*, 1997; Schmidt *et al*, 2001). Other reports have dealt with novel markers for the detection of micrometastases. Yonemura *et al* (2001) increased the sensitivity of detection to 62% by using a combination of CY and RT–PCR of MMP-7 mRNA. Schuhmacher *et al* (1999) used RT–PCR to show the relationship between the expression of E-cadherin mutation and metastasis to the peritoneum. But any assay using peritoneal wash is inferior in sensitivity and specificity when compared to the real-time RT–PCR for CEA mRNA described by Nakanishi *et al*. CEA is recently a standard molecular marker for the detection of gastric cancer micrometastasis. However, it is not expressed in all cases of peritoneal metastases, and very weakly in mesothelial cells, so that it is difficult to exclude false-positive or false-negative cases. This means that markers with greater sensitivity and specificity are needed to reduce misdiagnosis. It is very important to choose specifically expressed genes, which are most useful as markers of peritoneal dissemination, in order to exclude false-positive or false-negative readings. In other words, genes with a much higher expression in cancer cells than in mesothelial cells should be chosen. One of the novel markers selected by DNA microarray is DDC, which satisfies the above conditions.

DDC is responsible for the synthesis of the key neurotransmitters dopamine and serotonin, and is frequently expressed in neuroblastoma and small-cell carcinoma of the lung (North and Du, 1998; Gilbert *et al*, 1999). It is used for the differential diagnosis of neuroblastoma from other paediatric small round cell malignancies (Gilbert *et al*, 2000). DDC expression is also considered to be a marker for neuroendocrine differentiation in lung cancer cell lines (Jensen *et al*, 1990). Finally, it is also known as L-amino acid decarboxylase, which catalyses the synthesis of biogenic amines involved in different important functions, such as angiogenesis, cell proliferation, apoptosis and cell proliferation (Berry *et al*, 1996; Medina *et al*, 1999), which suggests that it plays an important role in gastric cancer progression.

In conclusion, our results show that DDC-specific RT–PCR is more sensitive than conventional CY or CEA–RT–PCR of peritoneal wash, so that this method may well be useful for the prediction of peritoneal recurrence in gastric cancer patients. In view of the correlation established by our study between PCR results and cancer recurrence, the use of DDC RT–PCR to predict peritoneal recurrence of gastric cancer is likely to be effective. A large-scale, long-term follow-up study is currently underway in our department to ascertain the actual rate of peritoneal recurrence in CY− and PCR-positive patients and to determine whether negative patients in fact remain disease-free. DDC is overexpressed in gastric cancer peritoneal dissemination, but the role of DDC in such dissemination is still unclear, so that further investigation is necessary to clarify it.
